# Inertial measurement units for the detection of the effects of simulated leg length inequalities

**DOI:** 10.1186/s13018-021-02212-z

**Published:** 2021-02-17

**Authors:** Hannah Lena Siebers, Jörg Eschweiler, Valentin M. Quack, Markus Tingart, Marcel Betsch

**Affiliations:** 1 Department of Orthopaedic Surgery, University Hospital RWTH Aachen, Pauwelsstr 30, 52074 Aachen, Germany; 2 University of Toronto Orthopaedic Sports Medicine Program (UTOSM), Women’s College Hospital, Toronto, Ontario Canada; 3 Department of Orthopaedics and Trauma Surgery, University Medical Center Mannheim of the University Heidelberg, Mannheim, Germany

**Keywords:** Gait analysis, Leg length difference, Joint angles, Compensation

## Abstract

**Background:**

Leg length inequalities (LLI) are a common condition that can be associated with detrimental effects like low back pain and osteoarthritis. Inertial measurement units (IMUs) offer the chance to analyze daily activities outside a laboratory. Analyzing the kinematic effects of (simulated) LLI on the musculoskeletal apparatus using IMUs will show their potentiality to improve the comprehension of LLI.

**Methods:**

Twenty healthy participants with simulated LLI of 0-4 cm were analyzed while walking with an inertial sensor system (MyoMotion). Statistical evaluation of the peak anatomical angles of the spine and legs were performed using repeated measurement (RM) ANOVA or their non-parametric test versions (Friedman test).

**Results:**

Lumbar lateral flexion and pelvic obliquity increased during the stance phase of the elongated leg and decreased during its swing phase. The longer limb was functionally shortened by higher hip and knee flexion, higher hip adduction, dorsiflexion, and lower ankle adduction. Finally, the shorter leg was lengthened by higher hip and knee extension, hip abduction, ankle plantarflexion, and decreased hip adduction.

**Conclusion:**

We found differing compensation strategies between the different joints, movement planes, gait phases, and amounts of inequality. Overall the shorter leg is lengthened and the longer leg is shortened during walking, to retain the upright posture of the trunk. IMUs were helpful and precise in the detection of anatomical joint angles and for the analysis of the effects of LLI.

## Introduction

Leg length inequalities (LLI) occur in up to 90% of the general population [1]. The majority of patients with smaller LLI (< 2 cm) are clinically asymptomatic [1] due to various compensation mechanisms of the musculoskeletal apparatus. On the other hand, in some patients, even minor LLI can lead to long-term detrimental effects such as premature osteoarthritis, low back pain, increased incidence of stress fractures, and running injuries [2, 3]. To this day, the exact amount of LLI that needs treatment to avoid long-term consequences is unknown.

Previous studies have examined the effects of LLI on the lower extremities, the pelvic position, or spinal posture. Here, it was shown that even LLI < 2 cm can lead to significant changes of the pelvic position and that LLI of > 2 cm can additionally lead to significant changes of the spinal posture in upright standing [4–7]. Similar results were found during walking, however, with smaller magnitude [8], because of various compensation mechanisms, e.g., shortening of the longer leg and lengthening of the shorter leg [3, 9]. The different effects of LLI showed the importance to investigate and analyze both the static as well as the dynamic response to LLI. Moreover, these previous studies have shown that it is relevant to study not only single isolated joints but also to focus on the kinematic chain of the musculoskeletal apparatus and how it is affected by LLI.

Wearable motion capture systems, e.g., inertial measurement units (IMUs), are of great interest for further research in this area. These devices can be used in a regular, non-laboratory environment to record patients’ gait and joint movements during daily and sports activities [10]. Furthermore, IMUs are reasonably priced, low-maintenance, easy-to-use, and allow for rapid analysis of the recorded data when combined with adequate software [11]. Like optoelectronic measurement systems, IMUs can measure kinematic data and anatomical angles in all three dimensions [12].

The purpose of this present study was to evaluate the feasibility of using IMUs to analyze the effects of (simulated) LLI on the musculoskeletal apparatus.

## Material and methods

### Participants

Included were 20 healthy participants, 8 men and 12 women, with a mean age of 25 ± 3 years, and a mean body mass index (BMI) of 22 ± 3 kg/m^2^. Subjects with LLI of ≥ 1 cm, any history of previous fractures of the lower extremities, pelvis, or spine, as well as any medical and neurological conditions (e.g., Parkinson and vascular disease, etc.) that influence gait and posture were excluded. The sample size of 20 volunteers was based on a sample size estimation (80% power, level of significance 5%, and an effect size of 0.4). All participants gave their written and oral consent as well as were given the option to discontinue their participation at any time. Institutional Review Board approval was obtained (EK 251/18).

### Measuring setup

**Fig. 1:**
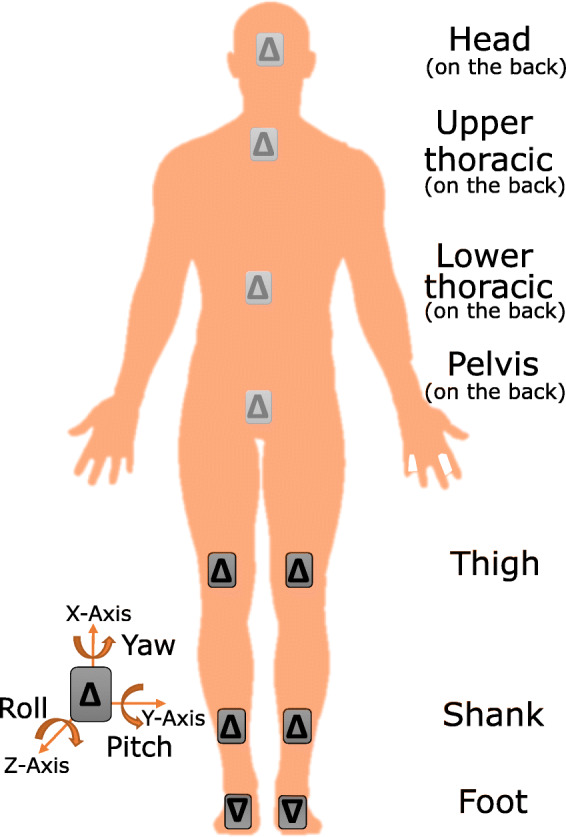
Measurement setup of the inertial sensors

Data were acquired with the IMU system MyoMotion (Noraxon USA Inc., Scottsdale, USA). A total of 10 sensors were positioned on previously determined anatomical landmarks and were attached with elastic straps or tape (Fig. 1). Based on the sensor data, the orientation angles (yaw (*x*), pitch (*y*), and roll (*z*)) of each body segment and the anatomical angles of the ankle, knee, hip, and spine (lumbar, thoracic, and cervical part) were calculated. The MyoMotion system allowed measurements with a sampling frequency of 100 Hz and an accuracy of 1° [12, 13].

### Measuring procedure

**Fig. 2:**
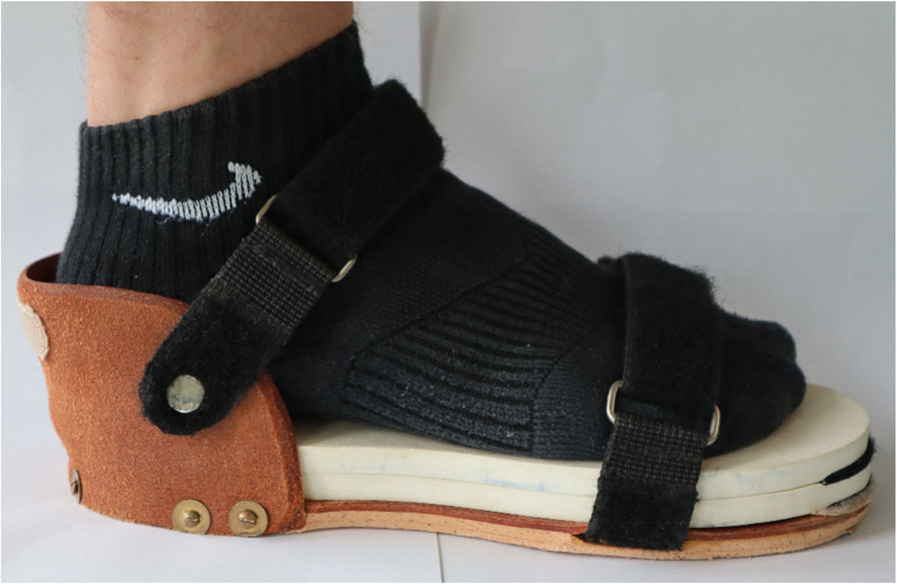
Custom-build sandal with added insoles to simulate LLI. The sandal was available in three different sizes to accommodate the subjects

Testing began by calibrating the IMUs. The IMUs were calibrated with the subjects standing in an upright standing position with the knees fully extended and the arms hanging to the sides. After the initial standing trial, all subjects were measured, a total of 9 times while walking at a self-selected speed. Walking began 2 m before and ended 2 m after a 10 m marked pathway in order to measure walking without any start or stop movements. First, the physiological gait was measured walking barefoot through a corridor, followed by measurements with increasing LLI. LLI were simulated by wearing a custom-built sandal on one leg (Fig. 2). The sole height of the sandal was controlled by various insoles of 1 cm height, from 1 cm to 4 cm. The sandal was randomly first worn on the left or right foot. We chose this method to simulate LLI since it is a common model for the simulation of LLI and because this method has been used successfully by our group before [3, 8, 9, 14].

### Data processing

For statistical analysis, the measured data were systematically processed and relevant parameters extracted. The initial and terminal ground contact of each step was evaluated by the contact detection algorithm of the MyoResearch Software (version 3.12, Noraxon USA Inc., Scottsdale, USA). Based on this information the *x*-axis (time-axis) was normalized to the gait cycle (in percent) and data were presented as angular values over one gait cycle. The MyoResearch Software interpolates one gait cycle to 100 points. For further analysis, the mean value over 4 strides was calculated and maxima and minima of the angles were determined during the stance and swing phase. The analysis was focused on the main movement planes of the spine, pelvis, hip, knee, and ankle. Therefore flexion/extension of hip and knee, dorsi/-plantarflexion of the ankle joint, ab-/adduction of the hip and ankle, pelvic obliquity, and lateral flexion of the spine were analyzed. Assuming the calibration position as the neutral-zero-position, each flexion and abduction was described with positive angles and the extension and adduction with negative values. Moreover, from a participant’s perspective, the lateral flexion of the spine to the right side and pelvic obliquity with a higher left hemipelvis was described with positive values and the opposite direction with negative values. The ankle ab- and adduction describes the rotation of the foot around the axis of the shank. For visualization, the angles over the gait cycle were calculated as a mean over all participants for each measurement condition and plotted with Matlab (MathWorks R2018b, MathWorks, Natick, MA, USA).

### Statistical analysis

The normal distribution of the dependent variables was evaluated using the Shapiro-Wilk test. To analyze the effects of simulated LLI on select parameters, repeated measurement analysis of variance (RM ANOVA) with partial eta squared for effect size, was used. If sphericity cannot be assumed, Greenhouse-Geisser correction was calculated. Bonferroni post hoc tests evaluated the test condition of LLI leading to significant changes compared to the physiological case. If the normal distribution was not assumed the Friedman-Test with Kendell’s *W* for the effect size and pairwise post hoc tests was applied. The level of significance was set at *p* < 0.05. Statistical analysis was performed using the software IBM® SPSS Statistics (IBM® SPSS Statistics v. 25, IBM Cooperation).

## Results

### Lateral flexion of the spine and pelvic obliquity

**Fig. 3:**
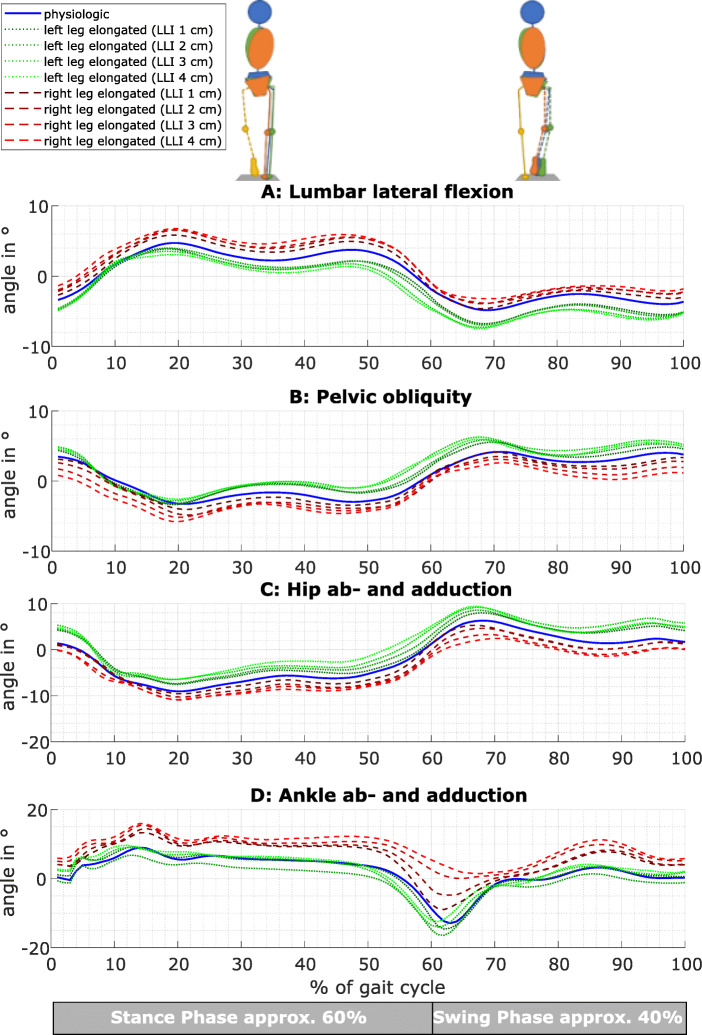
The movements of the right side/leg in the frontal plane are presented. They are calculated as a mean over all 20 participants. Physiological gait (blue) was compared to the movements with the right leg as the longer leg (red) and the shorter leg (green)

The lateral flexion of the cervical and thoracic spine was not significantly affected by any simulated LLI (Table 1). Physiologically, during the stance phase, the pelvis is higher on the side of the standing leg and the spine is also flexed to this side. During the swing phase, the movements are inverted (Fig. 3a and b, blue lines). Lengthening the right leg led to significantly increased lumbar lateral flexion during the stance phase and decreased flexion during the swing phase (Fig. 3a, red line; Table 1). Pelvic obliquity of the long (right) leg also increased during the stance phase and decreased during the swing phase with significantly higher/lower values compared to physiological gait for any simulated LLI (Fig. 3b, red line; Table 1). The effects on the short leg are in both cases inverted (Fig. 3a and b, green line; Table 1).

**Table 1:** Statistical evaluation with RM ANOVA or *Friedman-test* of frontal plane parameter

Parameter	Phase	Leg	Side	*P* value	Effect size	LLI with a significant difference to the physiological case	
						LLI	***P*** values
Max. cervical flexion	Stance	Long leg	Left	*0.355*	*0.061*		
			Right	*0.532*	*0.044*		
		Short leg	Left	*0.267*	*0.072*		
			Right	0.666	0.034		
	Swing	Long leg	Left	*0.132*	*0.098*		
			Right	*0.554*	*0.042*		
		Short leg	Left	*0.111*	*0.104*		
			Right	0.751	0.027		
Max. thoracal flexion	Stance	Long leg	Left	*0.441*	*0.049*		
			Right	0.197	0.079		
		Short leg	Left	0.530	0.037		
			Right	0.629	0.031		
	Swing	Long leg	Left	0.360	0.054		
			Right	0.245	0.072		
		Short leg	Left	0.326	0.061		
			Right	0.646	0.034		
Max. lumbar lateral flexion	Stance	Long leg	Left	0.008	0.202	1	0.045
			Right	< 0.001	0.447	≥ 2	< 0.001-0.008
		Short leg	Left	< 0.001	0.230	4	0.020
			Right	0.112	0.103	2	0.030
	Swing	Long leg	Left	< 0.001	0.449	All	< 0.001-0.003
			Right	< 0.001	0.295	2, 4	0.001
		Short leg	Left	< 0.001	0.276	All	0.002-0.036
			Right	0.001	0.297	2, 4	0.003
Max. pelvic obliquity	Stance	Long leg	Left	< 0.001	0.394	All	< 0.001-0.002
			Right	< 0.001	0.399	All	< 0.001-0.025
		Short leg	Left	< 0.001	0.322	1, 3, 4	0.001-0.035
			Right	< 0.001	0.671	All	< 0.001-0.006
	Swing	Long leg	Left	*< 0.001*	*0.494*	≥ 3	≤ 0.001
			Right	< 0.001	0.645	All	< 0.001-0.030
		Short leg	Left	< 0.001	0.418	All	< 0.001-0.010
			Right	< 0.001	0.561	All	≤ 0.001
Max. hip abduction	Stance	Long leg	Left	0.340	0.054		
			Right	*0.019*	*0.147*	*No*	
		Short leg	Left	< 0.001	0.358	All	< 0.001-0.046
			Right	< 0.001	0.288	≥ 3	0.006-0.010
	Swing	Long leg	Left	*< 0.001*	*0.431*	*≥ 2*	*< 0.001-0.019*
			Right	0.005	0.236	≥ 3	0.010-0.024
		Short leg	Left	< 0.001	0.466	All	< 0.001-0.026
			Right	< 0.001	0.521	≥ 3	< 0.001
Max. hip adduction	Stance	Long leg	Left	< 0.001	0.262	≥ 3	0.006-0.024
			Right	*< 0.001*	*0.283*	*≥ 3*	*≤ 0.001*
		Short leg	Left	0.099	0.112	3	0.027
			Right	0.006	0.169	No	
	Swing	Long leg	Left	0.002	0.195	3	0.045
			Right	*0.001*	*0.226*	*≥ 3*	*0.001-0.007*
		Short leg	Left	*0.013*	*0.160*	*3*	*0.014*
			Right	0.031	0.149	No	
Max. ankle abduction	Stance	Long leg	Left	< 0.001	0.256	3	0.008
			Right	*0.326*	*0.058*		
		Short leg	Left	*0.670*	*0.030*		
			Right	*0.300*	*0.061*		
	Swing	Long leg	Left	*0.005*	*0.186*	*≥ 3*	*0.005-0.037*
			Right	*0.033*	*0.132*	*No*	
		Short leg	Left	*0.518*	*0.041*		
			Right	*0.193*	*0.076*		
Max. ankle adduction	Stance	Long leg	Left	< 0.001	0.593	≥ 2	< 0.001
			Right	< 0.001	0.406	≥ 2	0.002-0.044
		Short leg	Left	0.517	0.034		
			Right	0.016	0.197	No	
	Swing	Long leg	Left	< 0.001	0.638	All	< 0.001-0.017
			Right	< 0.001	0.465	≥ 2	< 0.001-0.003
		Short leg	Left	0.487	0.037		
			Right	0.018	0.197	No

### Hip ab-/adduction

The maximum hip abduction of the longer right leg was significantly lower compared to the physiological gait during the swing phase for LLI of ≥ 3 cm. Moreover, the maximum hip adduction of the longer right leg was significantly higher compared to physiological gait for LLI of ≥ 3 cm during the stance and swing phase (Fig. 3c, red lines; Table 1). The maximum hip abduction of the shorter right leg was significantly higher compared to physiological gait during the stance and swing phase for LLI ≥ 3 cm (Fig. 3c, green lines; Table 1). The maximum hip adduction of the shorter right leg decreased significantly (Fig. 3c, green lines; Table 1).

### Ankle ab-/adduction

The maximum abduction of the left ankle (long side) was significantly higher compared to physiological gait for LLI of 3 cm during the stance and LLI of ≥ 3 cm during the swing phase. The maximum ankle adduction was significantly smaller for LLI ≥ 2 cm during the stance and swing phase (Fig. 3d, red lines; Table 1). The maximum ankle abduction of the short leg showed no significant changes during the stance or swing phases. The maximum ankle adduction of the short right leg increased significantly (Fig. 3d, green lines; Table 1).

### Hip flexion/extension

**Fig. 4:**
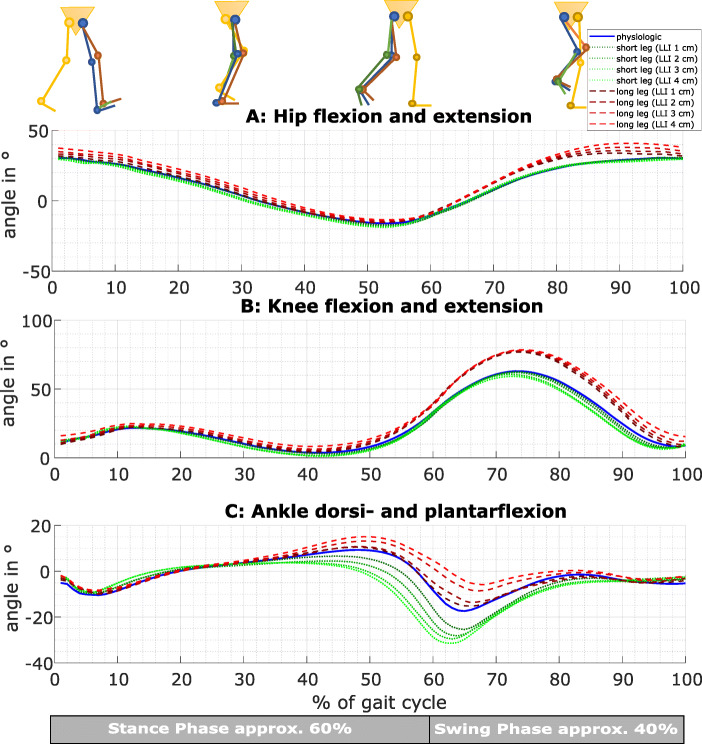
The movements of the right leg in the sagittal plane are presented. They are calculated as a mean over all 20 participants. Physiological movements (blue) are compared to the movements when this leg is lengthened (red) and when this leg is shortened (green)

The maximum hip flexion of the longer right leg was significantly higher compared to the physiological gait simulating any LLI during the stance phase and LLI ≥ 2 cm during the swing phase. Moreover, the maximum hip extension of the longer right leg was significantly lower for simulated LLI of ≥ 3 cm during the stance and swing phase (Fig. 4a red lines, Table 2). The maximum hip extension of the shorter right leg was significantly higher compared to physiological gait for any simulated LLI during the stance and swing phases (Fig. 4a green lines, Table 2).

**Table 2:** Statistic evaluation with RM ANOVA or *Friedman-test* of sagittal plane parameter

Parameter	Phase	Leg	Side	*p* value	Effect size	LLI with a significant difference to the physiological case	
						LLI	***p*** values
Max. hip flexion	Stance	Long leg	Left	< 0.001	0.664	≥ 2	< 0.001-0.004
			Right	< 0.001	0.784	All	< 0.001-0.011
		Short leg	Left	0.026	0.156	No	
			Right	*0.102*	*0.097*		
	Swing	Long leg	Left	< 0.001	0.806	All	< 0.001
			Right	*< 0.001*	*0.924*	*≥ 2*	*< 0.001-0.002*
		Short leg	Left	0.008	0.164	No	
			Right	0.037	0.141	No	
Max. hip extension	Stance	Long leg	Left	< 0.001	0.302	≥ 3	0.006-0.007
			Right	*< 0.001*	*0.386*	*≥ 3*	*< 0.001*
		Short leg	Left	*< 0.001*	*0.479*	*≥ 2*	*< 0.001-0.002*
			Right	< 0.001	0.603	All	< 0.001
	Swing	Long leg	Left	< 0.001	0.630	All	< 0.001-0.049
			Right	< 0.001	0.582	≥ 3	< 0.001
		Short leg	Left	*< 0.001*	*0.430*	*≥ 2*	*< 0.001-0.002*
			Right	< 0.001	0.586	All	≤ 0.001
Max. knee flexion	Stance	Long leg	Left	< 0.001	0.855	All	< 0.001
			Right	*< 0.001*	*0.876*	*≥ 2*	*< 0.001*
		Short leg	Left	< 0.001	0.727	All	< 0.001
			Right	< 0.001	0.749	All	< 0.001-0.027
	Swing	Long leg	Left	< 0.001	0.873	All	< 0.001
			Right	< 0.001	0.890	All	< 0.001
		Short leg	Left	< 0.001	0.351	≥ 3	0.005-0.011
			Right	< 0.001	0.462	4	0.014
Max. knee extension	Stance	Long leg	Left	*< 0.001*	*0.282*	*4*	*< 0.001*
			Right	*< 0.001*	*0.423*	*≥ 2*	*< 0.001-0.037*
		Short leg	Left	*< 0.001*	*0.351*	*≥ 3*	*< 0.001-0.002*
			Right	*< 0.001*	*0.555*	*≥ 2*	*< 0.001*
	Swing	Long leg	Left	< 0.001	0.418	4	0.009
			Right	< 0.001	0.713	≥ 3	< 0.001
		Short leg	Left	< 0.001	0.239	2, 4	0.017-0.034
			Right	0.001	0.252	2, 4	0.010-0.021
Max. ankle dorsiflexion	Stance	Long leg	Left	< 0.001	0.627	≥ 2	< 0.001-0.024
			Right	0.026	0.201	No	
		Short leg	Left	< 0.001	0.378	≥ 2	0.006-0.024
			Right	0.001	0.317	1-3	0.003-0.028
	Swing	Long leg	Left	*< 0.001*	*0.272*	*No*	
			Right	*< 0.001*	*0.357*	*4*	*0.014*
		Short leg	Left	*0.095*	*0.099*		
			Right	*0.012*	*0.160*	*2*	*0.007*
Max. ankle plantarflexion	Stance	Long leg	Left	*< 0.001*	*0.388*	*≥ 2*	*< 0.001-0.014*
			Right	< 0.001	0.466	≥ 3	< 0.001
		Short leg	Left	*< 0.001*	*0.459*	*≥ 2*	*< 0.001-0.014*
			Right	*< 0.001*	*0.360*	*All*	*< 0.001-0.019*
	Swing	Long leg	Left	< 0.001	0.618	All	< 0.001-0.014
			Right	< 0.001	0.558	≥ 2	< 0.001-0.043
		Short leg	Left	*< 0.001*	*0.465*	*≥ 2*	*< 0.001-0.005*
			Right	< 0.001	0.440	All	< 0.001-0.002

### Knee flexion/extension

The maximum knee flexion of the longer right leg was significantly higher compared to the physiologic gait for LLI ≥ 3 cm during the stance phase and for any LLI during the swing phase. Moreover, the minimum knee flexion of the longer leg was higher, and, therefore, showed a reduced extension compared to the physiological gait lengthening the right leg with ≥ 2 cm during stance and with ≥ 3 cm during the swing phase (Fig. 4b, red lines; Table 2). The maximum knee flexion of the short right leg was significantly lower during the stance phase for all LLI and during the swing phase for LLI of 4 cm. The minimum knee flexion of the shorter right leg was significantly lower simulating LLI ≥ 2 cm during the stance phase and LLI of 2 and 4 cm during the swing phase (Fig. 4b, green lines; Table 2).

### Ankle dorsiflexion/plantarflexion

The maximum ankle dorsiflexion on the long leg increased significantly with increasing LLI during the stance phase. The maximum ankle plantarflexion of the longer right leg was significantly reduced compared to the physiological gait for LLI ≥ 3 cm during the stance and LLI ≥ 2 cm during the swing phase (Fig. 4c, red lines; Table 2). The short right leg shows significantly reduced maximum dorsiflexion during the stance for LLI of 1-3 cm. The maximum plantarflexion of the short right leg was significantly higher for any simulated LLI during the stance and swing phase (Fig. 4c, green lines; Table 2).

### Summary

In Table 3, the effects of (simulated) LLI on the musculoskeletal apparatus are summarized.

**Table 3:** Summary of the effects on simulated LLI

	Ankle	Knee	Hip	Pelvis	Back
Long limb—stance phase	↑Dorsiflexion ↓Plantarflexion ↓Adduction	↑Flexion ↓Extension (increased minimal flexion)	↑Flexion ↓Extension ↑Adduction	↑Pelvic obliquity (lower on swing leg)	↑Lumbar lateral flexion (to longer side)
Long limb—swing phase	↓Plantarflexion ↑Abduction ↓Adduction	↑Flexion ↓Extension (increased minimal flexion)	↑Flexion ↓Extension ↓Abduction ↑Adduction	↓Pelvic obliquity (lower on swing leg)	↓Lumbar lateral flexion (to short side)
Short limb—stance phase	↓Dorsiflexion ↑Plantarflexion	↓Flexion ↑Extension (increased minimal flexion)	↑Extension ↑Abduction ↓Adduction	↓Pelvic obliquity (lower on swing leg)	↓Lumbar lateral flexion (to short side)
Short limb—swing phase	↑Plantarflexion	↑Extension (increased minimal flexion)	↑Extension ↑Abduction ↓Adduction	↑Pelvic obliquity (lower on swing leg)	↑Lumbar lateral flexion (to long side)

## Discussion

The purpose of this study was to analyze the effects of different simulated LLI on the kinematic chain of the musculoskeletal apparatus. This is the first study to use IMUs as a measuring technique to evaluate LLIs and their effects. Furthermore, this system can be used to study LLIs and their effects not just in a laboratory setting but also during daily and sports activities. We were able to show significant changes caused by LLI, whereat the compensation mechanics differ between the joints, movement planes, gait phases, side, and the amount of LLI.

Starting on the spine, we focused on the results in the frontal plane, with the lateral flexion as the main movement. We found only small movements and high variances caused by individual motion in the other planes. In previous studies, because of the use of different measuring systems, different parameters of the spinal posture were analyzed during static standing [4–7, 15] and while walking [8, 16]. However, using IMUs, we were able to analyze the motion of the cervical, thoracic, and lumbar spine. We did not find any significant changes in the motion of the cervical and thoracic spine between the different simulated LLI and physiological walking. In summary with our other results, LLI seems to be mostly compensated by the lower extremities, the pelvis, and the lower back. The development of the lumbar lateral flexion over the gait cycle (Fig. 3a), higher on the side of the standing leg, was comparable to the data reported by Needham et al. [16]. Moreover, the effects of LLI with significantly increased lumbar lateral flexion during the stance and decreased lumbar lateral flexion during the swing phase of the long leg were in accordance with the results reported by Needham et al. [16]. Additionally to Needham et al. [16], we found inverted effects on the short leg side.

The increased pelvic obliquity caused by LLI, lower on the side of the short leg, seems to be the biggest effect of LLI [4–8] and therefore pelvic obliquity is still used to diagnose and treat LLI [17]. While walking the pelvis was tilted, higher on the standing leg [16, 18]. Therefore, during the stance phase of the long leg, the maximum pelvic obliquity increased significantly [8, 9, 19] and then decreased significantly during its swing phase. In combination, while walking, the range of motion of the pelvis in the frontal plane was unaffected [16]. Following the biomechanical chain in the frontal plane, the hip motion was comparable with the pelvic motion (Fig. 3b, c) [18]. The long leg showed decreased hip abduction and increased hip adduction, whereas the short leg showed the contrary effects. The reduction of hip abduction on the elongated leg during the swing phase was contrary to the findings of Khamis et al. [3], whereas Zeitoune et al. [18] found higher adduction and lower abduction of the hip on the long leg compared to the short leg. Moreover, Resende et al. [9] presented increased hip abduction for the short leg during the stance phase in comparison to our results.

In the sagittal plane, the hip flexion increased on the long leg to functionally shorten the leg [3, 9, 19, 20]. Moreover, we analyzed the maximum hip extension, which decreased in the long leg and increased in the short leg. Similar results, increased flexion [3, 9, 19, 20] and decreased extension of the long leg, and increased extension of the short leg [3, 9, 19], were found for the knee motion. The ankle joint of the long leg showed decreased plantarflexion and increased dorsiflexion during the stance phase [19]. The short leg showed the contrary effects with increased plantarflexion [3, 9, 19] and decreased dorsiflexion [19]. In the frontal plane, the ab- and adduction of the ankle was only affected in the long limb, showing higher ab- and significantly lower adduction. Khamis et al. [3] described a similar effect showing the increased external rotation of the foot in the long leg. In combination, the purpose of the compensation mechanisms seems to functionally shorten the longer limb and lengthen the shorter limb, as previously described [3], with fewer effects in the short leg [9, 19].

Most of our results were in accordance with previous studies, which underline the capability of the IMU system for musculoskeletal analysis. One exception was the decreased hip abduction of the long leg compared to the findings of Zeitoune et al. [18], but contrary to the findings of Khamis et al. [3]. Moreover, small differences concerning the trend in the angle diagrams were supposed to result predominantly by differences of biomechanical models used for angle estimation and from different measurement techniques [21]. The limitations caused by drift effects of the IMUs were reduced because of a short walking task as a pendulum motion [21]. Other differences to previous studies are caused by the statistical analysis, where we evaluated peak angle values during the stance and swing phase, in contrast to the analysis of the anatomical angles during specific gait events [3, 19].

To analyze the effect of LLI, we decided to measure healthy participants with different simulated LLI, instead of patients with anatomic LLI. Compared to previous studies, this was an acceptable method [3, 8, 9, 14]. Analyzing participants with simulated LLI allows the personal comparison between physiological and pathological gait, the effects of different amounts of LLI, and bipedal comparison. But the simulation of LLI with the custom-build sandal is still a limitation. Walking with the sandal had an influence on the roll-over movement, especially compared to barefoot walking [22]. Moreover, we analyzed only the acute effect, without compensation strategies generated over a time period. The simulation of LLI with different sole-height between the legs is based on the therapy of mild LLI with orthopedic insoles and not comparable with the location of “real” LLI in the tibia or femur. But as described, with this method, we were able to show the possibilities of the measurement technique and the method. There are a lot of questions that have to be answered in the discussion of the correct therapy for LLI. As a first step, we attached 10 IMUs to the body, to analyze the link chain from the lower extremities over the pelvis and spine up to the head. For further analysis, we are able to exceed the measurement system easily with up to 16 sensors, which will additionally allow analyzing upper extremity movements. Especially during dynamic motion upper extremities movement is also affected because of LLI [23] and therefore needs to be evaluated in addition. With this setup, it will be able to evaluate the correlation effects of the different parts/joints of the biomechanical chain. Following this, possible different compensation strategies can be evaluated, explaining the differences in the effect of LLI on different people. In another step, IMUs can be used in the analysis of the biomechanical effects of “real” LLI and their treatments in patients. Analyzing patients, the calibration position for the IMUs must be adapted to overcome errors because of the affected standing position. One solution could be the calibration in a balanced standing position with insoles, controlled by posture analysis systems [6–8].

## Conclusion

Our results showed that (simulated) LLI lead to gait asymmetry and compensation strategies of the musculoskeletal apparatus, mainly on the legs, the pelvis, and the lumbar spine, in the sagittal and frontal plane (Table 3). Participants with (simulated) LLI tried to functionally shorten the long leg and lengthen the short leg, whereas the magnitude of compensation differed between long and short leg, the gait phases, the joints, planes, and the amount of LLI. This is the first study to demonstrate that the effects of LLI can be measured with an IMU system, which is a user-friendly option to analyze motion during daily activities.

## Data Availability

The datasets used and/or analyzed during the current study are available from the corresponding author on reasonable request.

## Abbreviations

LLI: Leg length inequality
IMU: Inertial measurement unit
BMI: Body mass index
RM ANOVA: Repeated measurement analysis of variance
